# Plasma Lipidomics Reveals Insights into Anti-Obesity Effect of *Chrysanthemum morifolium* Ramat Leaves and Its Constituent Luteolin in High-Fat Diet-Induced Dyslipidemic Mice

**DOI:** 10.3390/nu12102973

**Published:** 2020-09-29

**Authors:** Jong Cheol Shon, Won Cheol Kim, Ri Ryu, Zhexue Wu, Jong-Su Seo, Myung-Sook Choi, Kwang-Hyeon Liu

**Affiliations:** 1 Environmental Chemistry Research Group, Korea Institute of Toxicology, Jinju 52834, Korea; sleier7640@naver.com (J.C.S.); jsseo@kitox.re.kr (J.-S.S.); 2 College of Pharmacy and Research Institute of Pharmaceutical Sciences, Kyungpook National University, Daegu 41566, Korea; wk3012@naver.com (W.C.K.); wuzhexue527@gmail.com (Z.W.); 3 Research Institute of Eco-Friendly Livestock Science, Institute of Green-Bio Science and Technology, Seoul National University, Pyeongchang 25354, Korea; sangsang0119@gmail.com; 4 Center for Food and Nutritional Genomics Research, Kyungpook National University, Daegu 41566, Korea

**Keywords:** *Chrysanthemum morifolium* Ramat leaves, obesity, lipidomics, liquid chromatography tandem mass spectrometry, phospholipid, sphingolipid

## Abstract

The *Chrysanthemum morifolium* Ramat (CM) is widely used as a traditional medicine and herbal tea by the Asian population for its health benefits related to obesity. However, compared to the flowers of CM, detailed mechanisms underlying the beneficial effects of its leaves on obesity and dyslipidemia have not yet been elucidated. Therefore, to investigate the lipidomic biomarkers responsible for the pharmacological effects of CM leaf extract (CLE) in plasma of mice fed a high-fat diet (HFD), the plasma of mice fed a normal diet (ND), HFD, HFD plus CLE 1.5% diet, and HFD plus luteolin 0.003% diet (LU) for 16 weeks were analyzed using liquid chromatography-tandem mass spectrometry (LC-MS/MS) combined with multivariate analysis. In our analysis, the ND, HFD, CLE, and LU groups were clearly differentiated by partial least-squares discriminant analysis (PLS-DA) score plots. The major metabolites contributing to this differentiation were cholesteryl esters (CEs), lysophosphatidylcholines (LPCs), phosphatidylcholines (PCs), ceramides (CERs), and sphingomyelins (SMs). The levels of plasma CEs, LPCs, PCs, SMs, and CERs were significantly increased in the HFD group compared to those in the ND group, and levels of these lipids recovered to normal after administration of CLE or LU. Furthermore, changes in hepatic mRNA expression levels involved in the Kennedy pathway and sphingolipid biosynthesis were also suppressed by treatment with CLE or LU. In conclusion, this study examined the beneficial effects of CLE and LU on obesity and dyslipidemia, which were demonstrated as reduced synthesis of lipotoxic intermediates. These results may provide valuable insights towards evaluating the therapeutic effects of CLE and LU and understanding obesity-related diseases.

## 1. Introduction

Obesity, one of the major worldwide health problems in recent years, is usually caused by an imbalance in food intake and energy expenditure, with overconsumption of energy intake or decrease in physical activity. Among the various models of inducing obesity in animals, the high-fat diet (HFD)-induced obesity model alters diverse biochemical factors, such as insulin, eventually leading to abnormal lipid metabolism. HFD-induced obesity is characterized by abnormally high levels of blood lipids, such as cholesterol, low-density lipoprotein (LDL), and triacylglycerol (TAG), leading to progressive weight gain in various tissues of the body (e.g., liver and fat tissues) due to excess fat accumulation [1,2]. It is also associated with an increase in the prevalence and risk of metabolic syndrome-related diseases, including type 2 diabetes (T2D), dyslipidemia, cardiovascular diseases (CVD), hypertension, and fatty liver [3,4]. Especially, the development of obesity by HFD indicates that it is pathophysiologically very similar to obesity in humans [5,6].

*Chrysanthemum morifolium* Ramat (CM) has been used for almost a hundred years in the form of a traditional medicine, beverage, and herbal tea in many Asian countries, including Japan, China, Thailand, and Korea, due to its health benefits related to inflammation, hypertension, and arteriosclerosis. The main bioactive components in the flowers of CM are flavonoids and phenolic acids, which are composed of chlorogenic acid, acacetin, apigenin, and luteolin, along with its glucoside form [7]. Previous studies have shown that CM and its components possess various biological functions, including antioxidant [8], anti-inflammatory [9], anti-tumorigenic [10], and cardiovascular-protective functions [11]. Moreover, the CM flower extract has been shown to prevent hyperlipidemic fatty liver disease by increasing hepatic peroxisome proliferator-activated receptor (PPAR)-α expression in hyperlipidemic fatty liver disease caused by high-fat milk [12]. Such beneficial effects have also been reported in HFD-induced obese mice [13] and an alloxan-induced diabetes mellitus model [14]. Almost all the studies conducted on the efficacy of CM related to health-promoting effects have been focused on its flowers. However, although not used as a traditional medicine, unlike flowers, the leaves of CM have a large amount of potential bioactive components, such as flavonoids, galuteolin, quercetin, and chlorogenic acid [15,16]. Consequently, the leaves can be used for medicinal purposes because of their high content of bioactive components. Furthermore, a recent study revealed that weight gain, fat deposition, and plasma lipid levels were significantly reduced by the administration of CM leaf extract (CLE) in HFD-fed mice [17]. However, apart from the previous approach based on the major lipids, such as cholesterol, lipoprotein, and TAG, detailed studies on lipid profiles, including changes in individual lipid species, towards the pharmacological effects of CLE in obese mice have rarely been described.

Modern lipidomics technology based on mass spectrometry provides significant insights into the metabolism and alterations in lipids through the identification and quantification of individual lipid species in a broad range of lipids [18]. This technology has been used extensively in biomedical research to evaluate toxicity and pharmacological properties of drugs [19], to discover potential biomarkers for disease states [20], and to understand the pathological mechanism of the metabolic syndrome [21]. Previous studies have shown that changes in specific lipid profiles and composition, such as ceramides and sphingomyelin species, are closely related to obesity-related diseases and metabolic disorders [22,23]. In addition, these techniques have also been used to explore the pharmacological effects of herbal medicines, such as *Cyclocarya paliurus* [24] and *Camellia sinensis* leaves [25], and *Panax ginseng* radix [26].

Thus, in this study, we conducted lipidomics analysis in HFD-induced metabolic disorders representing obesity and dyslipidemia as a pathology model. In addition, we also investigated the pharmacological effects of CLE and luteolin, which are closely related to the physiological functions and quality of CM [27], and its mechanisms against HFD-induced dyslipidemia through alterations of plasma lipid metabolites using the technique of liquid chromatography coupled with tandem mass spectrometry (LC-MS/MS).

## 2. Materials and Methods

### 2.1. Chemicals and Reagents

Methanol, isopropanol, and water were purchased from Merck (LC-MS grade, Darmstadt, Germany). Ammonium acetate, chloroform, methyl-*tert*-butyl ether (MTBE), butylhydroxytoluene (BHT), and TAG (15:0/15:0/15:0) were purchased from Sigma-Aldrich (St. Louis, MO, USA). Lysophosphatidylcholine (LPC 17:1), lysophosphatidylethanolamine (LPE 17:1), phosphatidylcholine (PC 17:0/14:1), phosphatidylethanolamine (PE 17:0/14:1), diacylglycerol (DAG 8:0/8:0), sphingomyelin (SM d18:1/12:0), ceramide (CER d18:1/12:0), and cholesteryl ester (CE 15:0) were obtained from Avanti Polar Lipids (Alabaster, AL, USA). The lipid standards mentioned above were used as internal standards for semi-quantification of each lipid.

### 2.2. Preparation of Chrysanthemum morifolium Ramat Leaf Extract

**Figure 1:**
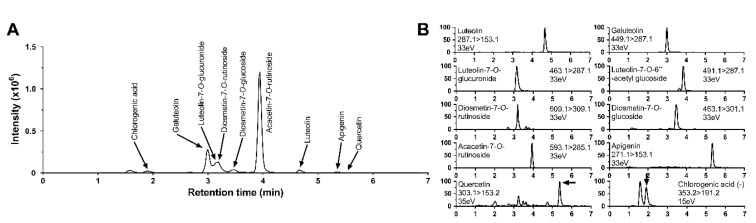
The total ion chromatogram (**A**) and selected reaction monitoring chromatogram (**B**) of individual flavonoids from *Chrysanthemum morifolium* Ramat leaf extracts (CLE).

CM leaves (Haihang Industry, Jinan, China) were washed, crushed to 20 mesh, and extracted by adding 10 volumes of 70% ethanol to 10 kg CLE in an extraction vessel. The extraction was performed at 65–70 °C in the roof of the pot, which had a condenser. Vapor was condensed and returned back to the pot, which was recirculated three times, and each time-period lasted for 2 h. The extracted CLE liquid was subsequently concentrated and spray-dried at 120 °C to 80 mesh. The yield obtained was 10%. The contents of flavonoids in the CLE were analyzed using liquid chromatography-tandem mass spectrometry (LC-MS/MS) as described in previous papers [28,29,30]. The contents (mg/g dry weight) of ten flavonoids in CLE were as follows: luteolin, 0.057; galuteolin, 0.47; luteolin-7-*O*-glucuronide, 0.28; luteolin-7-*O*-6-acetyl glucoside, 0.0047; diosmetin-7-*O*-rutinoside, 0.056; diosmetin-7-*O*-glucoside, 0.055; acacetin-7-*O*-rutinoside, 1.7; apigenin, 0.0062; quercetin, 0.0032; chlorogenic acid, 0.19 (Figure 1).

### 2.3. Animal Experiments

Male C57BL/6J mice (4-week-old) were obtained from the Jackson Laboratory (Bar Harbor, ME, USA). All the mice were individually housed under constant temperature (24 °C) and a 12 h light/dark cycle, fed a normal chow diet for a one-week acclimation period, and subsequently, randomly divided into four groups. The mice in the different groups were fed a normal diet (ND, 12% kcal from fat, *n* = 6), high-fat diet (HFD, 45% kcal from fat, *n* = 6), HFD with 1.5% CLE (*w*/*w*) (*n* = 6), and HFD with 0.003% luteolin (*w*/*w*) (LU, *n* = 6) for 16 weeks. The mice were given free access to food and distilled water, and food intake and body weight were measured weekly. At the end of the mice experimental period, all the mice were anesthetized with isoflurane after 12 h of fasting. Blood samples were collected from the inferior vena cava in heparin-coated tubes, and plasma samples were obtained by centrifuging blood at 4000× *g* for 15 min at 4 °C. The liver, epididymal white adipose tissue (WAT), perirenal WAT, retroperitoneal WAT, mesentery WAT, subcutaneous WAT, interscapular WAT, interscapular brown adipose tissue, and skeletal muscle were removed, weighed, and immediately frozen in liquid nitrogen and stored at 80 °C. Animal studies were performed using protocols approved by the Kyungpook National University Industry Foundation (Approval No. KNU-2016-49).

### 2.4. Lipid Extraction

Lipids were extracted from mouse plasma according to the Matyash method with slight modifications [31]. In brief, a 50 µL internal standard (IS) mixture (LPC 17:1 400 ng/mL; LPE 17:1 400 ng/mL; PC 17:0/14:1 400 ng/mL; PE 17:0/14:1 400 ng/mL; DAG 8:0/8:0 40 ng/mL; SM d18:1/12:0 400 ng/mL; CER d18:1/12:0 40 ng/mL; CE 15:0 4 µg/mL; TAG 15:0/15:0/15:0 40 ng/mL) was transferred to a 2 mL Eppendorf tube and dried. Later, 5 µL plasma, 300 µL cold methanol, 100 µL water, and 1 mL MTBE along with 0.1% BHT were added to the tube. The mixture was shaken for 1 h at room temperature. Next, phase separation was induced by adding 250 µL water and incubating for 10 min at room temperature. After centrifugation (14,000× *g* for 15 min at 4 °C), the two phases were separately collected in 1.5 mL tubes. The upper (220 µL) and lower (110 µL) fractions were pooled and dried in a vacuum centrifuge (LABCONCO, Kansas City, MO, USA) for lipid profiling. The dried lipid extracts were reconstituted in 100 µL chloroform/methanol (1:9, *v*/*v*) prior to lipidomics analysis. Using the above method, hepatic lipids were extracted from the lyophilized samples (10 mg).

### 2.5. Lipidomics Study

Analysis of lipid extracts was performed using a high-performance liquid chromatography (HPLC) apparatus coupled to a Shimadzu 8040 tandem mass spectrometer (Shimadzu Corporation, Kyoto, Japan), based on a previously reported method with some modifications [32,33]. Semi-quantitative analysis of the extracted lipids from the plasma of mice was carried out using a Kinetex C18 column (100 × 2.1 mm, 2.6 μm, Phenomenex, Torrance, CA, USA) with a flow rate of 200 μL/min in positive mode. Mobile phase A was 10 mM ammonium acetate in water:methanol (1:9, *v*/*v*) and mobile phase B was 10 mM ammonium acetate in methanol:isopropanol (1:1, *v*/*v*). To achieve the best separation of the lipids, gradient elution was conducted as follows: 30% B (0 min), 95% B (15 min), 95% B (20 min), and 30% B (20–25 min). Each lipid quantitation was performed by selected reaction monitoring (SRM) of the precursor ions, ([M + H]^+^ or [M + NH_4_]^+^), and the related product ion for each lipid. To calculate the concentration of each target lipid species, the ratio of target analyte and internal standard (IS) was multiplied by the concentration of one specific IS representing each lipid species based on single-point calibrations [34,35,36]. IS and SRM transition ions for each lipid class are described in a previously published paper [33].

### 2.6. Hepatic Gene Expression Analyses

Total RNA from the liver was extracted from three representative samples in each group according to the manufacturer’s instructions (Invitrogen Life Technologies, Grand Island, NY, USA). The libraries of mRNA sequences were prepared as paired-end reads with a length of 100 bases using the TruSeq RNA Sample Preparation Kit (Illumina, CA, USA). After cDNA synthesis, the quality of these cDNA libraries was evaluated employing the Agilent 2100 Bioanalyzer (Agilent, CA, USA), and then sequenced as paired-ends using Illumina HiSeq2500 (Illumina, CA, USA). Quality control was performed using FastQC v. 0.11.2. Transcripts were assembled in Cufflinks v2.2.1 with TopHat Aligner [17,37]. Gene expression levels were estimated as fragments per kilobase of transcript per million mapped reads. Normalization factors were calculated using the differentially expressed genes (DEGES)/Empirical analysis of digital gene expression data in R (edgeR) method. Differentially expressed genes were identified based on a q-value threshold of less than 0.05. The RNA-sequence data is available at the National Center for Biotechnology Information (NCBI)’s Gene Expression Omnibus Database (http://www.ncbi.nlm.nih.gov/geo/): accession number GSE124777.

### 2.7. Data Processing and Statistical Analyses

To confirm stability and reproducibility of the mass spectrometric analysis, a quality control (QC) sample [National Institute of Standards and Technology (NIST) Standard Reference Material (SRM 1950 plasma)] was loaded at regular intervals (ten samples) [38,39]. Unsupervised principal component analysis (PCA) and supervised partial least squares-discriminant analysis (PLS-DA) multivariate analyses were used to construct pattern recognition models based on plasma lipidomics data with Pareto scaling, to explore the extent of differences among the four groups using the software SIMCA-P+ (version 13.0, Umetrics, Umea, Sweden). All data were reported as mean ± standard deviation (SD). Statistically significant differences between all the groups were calculated by one-way analysis of variance (ANOVA) and Tukey’s multiple comparison test using Statistica 7 (StatSoft Inc., Tulsa, OK, USA).

## 3. Results and Discussion

### 3.1. Animal Characteristics

**Figure 2:**
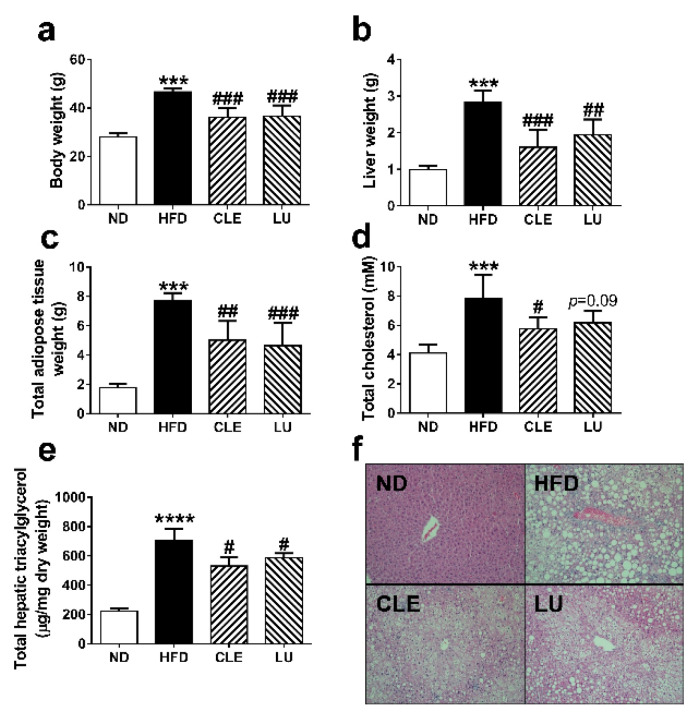
Effect of *Chrysanthemum morifolium* Ramat leaf extracts (CLE; 1.5%) and luteolin (LU; 0.003%) on body weight (**a**), liver weight (**b**), total adipose tissue weight (**c**), total cholesterol level (**d**), total hepatic triacylglycerol contents (**e**), and hepatic morphology (original magnification × 100) (**f**) in C57BL/6J mice fed a high-fat diet (HFD). The data are shown as mean ± standard deviation (SD) (*n* = 6). *, # different letters indicate significant difference compared with normal diet (ND) group (*) and HFD group (#), as determined by Tukey’s multiple comparisons test. (*** *p* < 0.001, **** *p* < 0.0001, # *p* < 0.05, ## *p* < 0.01, ### *p* < 0.001).

Male C57BL/6J mice were fed an ND, HFD, HFD + CLE, and HFD + LU for 16 weeks. Changes in biological characteristics of these mice are shown in Figure 2. The values of body weight, liver weight, total adipose tissue weight, and total cholesterol were 28.1 ± 1.4 g, 1.0 ± 0.1 g, 1.8 ± 0.2 g, and 4.1 ± 0.6 mmol/L in the ND group, respectively; however, due to administration of HFD, these levels were significantly elevated to 46.6 ± 1.5 g, 2.8 ± 0.3 g, 7.7 ± 0.6 g, and 7.8 ± 1.7 mmol/L respectively, indicating that the obese mouse model was well-established. In contrast, supplementation with CLE or LU significantly lowered body weight (*p* < 0.001), liver weight (*p* < 0.01), and total adipose tissue weight (*p* < 0.01) compared to that in the HFD group. Recently, CLE was shown to ameliorate insulin resistance, thermogenesis, and energy expenditure along with a decrease in weight gain in white adipose tissue [17]. Liver histological analysis using Hemotoxylin and Eosin (H&E) staining was employed to evaluate the preventive effects of CLE on HFD-induced obese mice (Figure 2f). The livers of mice in the ND group did not exhibit lipid deposition and signs of steatosis, whereas those in the HFD group displayed accumulation of lipid droplets and developed hepatic steatosis. However, the CLE- or LU-treated groups exhibited a significant reduction of hepatic lipid droplets. While LU also decreased the number of lipid droplets in the liver as CLE, this effect tended to be less (Figure 2f). The hepatic TAG contents in each group were calculated by summing the contents of all quantified TAGs. The hepatic TAG contents in the ND, HFD, CLE, and LU groups were 222.3 ± 21.2, 707.0 ± 76.5, 531.6 ± 57.2, and 587.2 ± 31.0 μg/mg, respectively (Figure 2e). As expected, the hepatic TAG contents in the CLE and LU groups were lower than those in the HFD group (*p* < 0.01), and most individual TAG species in CLE tended to decrease slightly more than those in the LU group (*p* > 0.05) (Supplementary Figure S1). The more effective decrease of lipid droplet number and total hepatic triacylglycerols in the CLE treatment group compared to the LU treatment may be due to bioactive compounds such as quercetin and chlorogenic acid present with luteolin in CM leaves. Energy expenditure was significantly increased during a 12 h light/dark cycle after CLE and LU treatment when compared with the HFD group. Considering the suppressed hepatic lipogenesis and enhanced energy expenditure by CLE and LU treatment [17], it was evident that CLE and LU have a potential to attenuate obesity in mice.

### 3.2. Multivariate Statistical Analysis of Mice Plasma Lipid Levels

**Figure 3:**
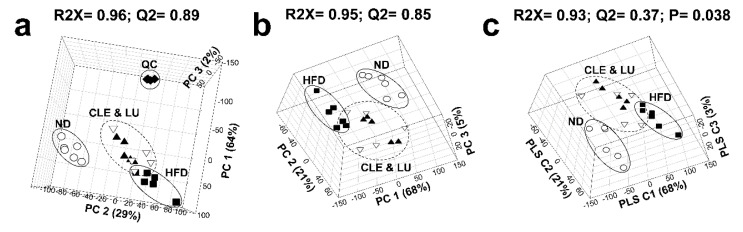
Principal component analysis (PCA) three-dimensional (3D) score plots (**a**), PCA 3D score plots excluding quality control (QC) (**b**), and partial least-squares discriminant analysis (PLS-DA) 3D score plots (**c**) based on the serum lipidomic profiling among four groups. (Circle (○): normal diet (ND); Box (■): high-fat diet (HFD); Inverted triangle (▽): HFD plus *Chrysanthemum morifolium* Ramat leaf extract 1.5% diet (CLE); Triangle (▲): HFD plus luteolin 0.003% diet (LU); Diamond (◆): Quality control (QC)).

Representative pooled mice plasma samples were used to identify lipids therein. Approximately 140 individual lipid molecular species covering 9 lipid classes, comprising 16 LPC, 27 PC, 6 LPE, 11 PE, 41 TAG, 5 DAG, 14 SM, 14 CE, and 6 CER, were identified from pooled plasma samples of the mice [26]. The lipid profiles thus obtained from the ND, HFD, CLE, and LU groups were analyzed by multivariate statistical analysis to identify the extent of differences among the four groups. In the PCA score plot (Figure 3a), QC samples (NIST SRM1950 standard blood plasma, [40]) were tightly clustered, suggesting that the mass spectrometric analysis data has excellent stability and reproducibility. Performance of the constructed principal component analysis (PCA) and partial least-squares discriminant analysis (PLS-DA) model was assessed by fitness (*R*^2^) and predictability (*Q*^2^), and the performance levels of the good models are higher than 50%. The significance of the PLS-DA model was evaluated using cross-validated analysis of variance (CV-ANOVA) [41]. Each group showed a clear distinction in the PCA (*R*^2^X = 95%, *Q*^2^ = 85%) and PLS-DA (*R*^2^X = 93%, *Q*^2^ = 37%, *p* = 0.038) score plots with a good model quality. Clusters of lipid species from the CLE and LU groups were observed as separate clusters from those of the HFD group (Figure 3b,c).

### 3.3. Effect of a High-Fat Diet on Mice

**Figure 4:**
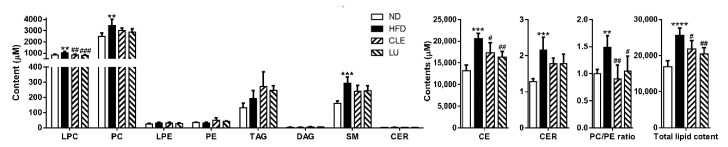
Overview of the effects of *Chrysanthemum morifolium* Ramat leaf extracts and luteolin on the serum lipidome in obese mice. The total lipid contents are expressed as the sum of contents of all identified lipids in mice plasma. Lipid classes are indicated on the *x*-axis: LPC, lysophosphatidylcholine; PC, phosphatidylcholine; LPE, lysophosphatidylethanolamine; PE, phosphatidylethanolamine; TAG, triacylglycerol; DAG, diacylglycerol; SM, sphingomyelin; CER, ceramide; CE, cholesteryl ester. The data are shown as mean ± standard deviation (SD) (*n* = 6). *, # different letters indicate significant difference compared with the normal diet (ND) group (*) and high-fat diet (HFD) group (#), as determined by Tukey’s multiple comparisons test. CLE: HFD plus *Chrysanthemum morifolium* Ramat leaf extract 1.5% diet; LU: HFD plus luteolin 0.003% diet (** *p* < 0.01, *** *p* < 0.001, **** *p* < 0.0001, # *p* < 0.05, ## *p* < 0.01, ### *p* < 0.001).

The sum (total) levels of the lipid species quantified in the plasma samples of mice were significantly elevated by HFD, and the lipid species contributing to these changes were LPC, PC, CE, SM, and CER. In contrast, the levels of LPE, PE, TAG, and DAG did not differ between the groups (Figure 4). The levels of 13 of 14 CE species, 12 of 27 PC species, 12 of 16 LPC species, all SM species, and 5 of 6 CER species were significantly increased by HFD, as shown in Supplementary Table S1. Similar to our results, accumulation of CE, PC, LPC, and sphingolipids (CER and SM) in plasma of HFD-induced obese and dyslipidemic mice has been reported [23,24,26,42,43,44]. The PC/PE ratio is a well-known marker associated with health and disease and regulates a variety of cellular processes [45]. Recently, an increase in these indicators has also been observed in the plasma of dyslipidemic and diabetic mice [24] and WAT macrophages in leptin-deficient (*ob/ob*) mice [46]. Consistent with this result, our results also showed a significant elevation in the levels of PC and PC/PE ratio in the HFD group compared with those in the ND group (Figure 4).

### 3.4. Effect of Chrysanthemum morifolium Ramat leaf Extract (CLE) and Luteolin (LU) in Mice

**Figure 5:**
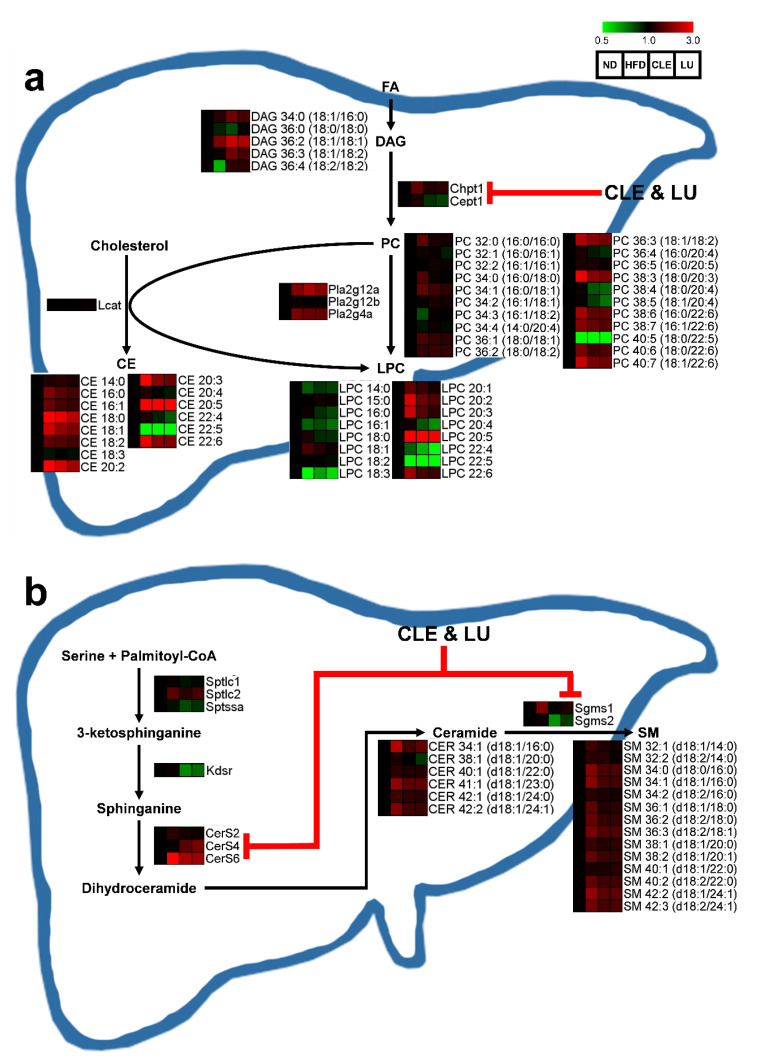
Scheme of the phospholipid synthesis (**a**) and sphingolipid synthesis (**b**) by which *Chrysanthemum morifolium* Ramat leaf extracts and luteolin regulates serum lipids and related hepatic gene-expression. The features of these compared to the ND group are color-coded by row, with red indicating high levels and green indicating low levels. ND: normal diet; HFD: high-fat diet; CLE: HFD plus *Chrysanthemum morifolium* Ramat leaf extract 1.5% diet; LU: HFD plus luteolin 0.003% diet; FA: fatty acid; DAG: diacylglycerol; PC: phosphatidylcholine; LPC: lysophosphatidylcholine; CE: cholesteryl ester; CER: ceramide; SM: sphingomyelin.

We found that the sum (total) levels of LPC, PC, CE, SM, and CER were elevated in the HFD group compared to those in the ND group. Compared with the HFD group, CLE or LU treatment significantly reduced the sum total of the LPC and CE (*p* < 0.05), and also showed a tendency to decrease the SM and CER (*p* = 0.08) (Figure 4). However, even though CLE or LU had little effect on altering the sum total of the PC, the PC/PE ratio completely recovered to that of the ND group (Figure 4). The level of PC can be modulated by converting PE to PC by phosphatidylethanolamine *N*-methyltransferase (PEMT) as well as the Kennedy pathway that comprises Chpt1 and Cept1. The depletion of PC content by knockout of PEMT led to an increase in energy expenditure, weight loss, and insulin sensitivity, thereby preventing HFD-induced obesity and atherosclerosis [47,48,49]. Importantly, significantly higher hepatic expression of Chpt1 was observed in HFD-induced obese mice, however, CLE treatment significantly suppressed both the levels of Chpt1 and Cept1, while LU treatment significantly reduced only Cept1 (Figure 5a). In contrast, no statistically significant change in the hepatic expression of PEMT was noted between the groups (data not shown). Thus, CLE or LU treatment appears to have an inhibitory effect on the Kennedy pathway rather than through the PEMT pathway.

LPC is generated from the PC either via deacylation by phospholipase A_2_ (PLA_2_) isozymes or conversion by lecithin-cholesterol acyltransferase (LCAT) and is well known to be one of the major lipotoxic intermediates that promotes metabolic diseases through specific cell signaling, such as endoplasmic reticulum (ER) stress, chronic inflammation, apoptosis, and necrosis [50,51,52]. Consistent with our results, many previous studies have reported that the levels of most LPCs, including LPC 18:0, LPC 18:1, LPC 20:3, and LPC 22:6, were significantly increased in plasma of obese mice [26,42,44]. In addition to LPC, high plasma levels of CE are strongly recognized as a key marker to assess the risk and prediction of CVD [53,54]. Several studies have demonstrated that accumulation of high levels of CEs in plasma was observed in HFD-streptozotocin-induced diabetic (*db/db*) mice and HFD-induced obese mice, which are consistent with our findings [23,24,26,42]. In our study, one of the interesting findings was that significantly reduced levels of most LPC and CE were observed after CLE or LU treatment. However, no significant changes in the hepatic gene expressions, including LCAT, PLA_2_ isozymes, and lysophosphatidylcholine acyltransferase (LPCAT) isozymes that are involved in synthesis of these lipids, were observed between all the groups (*p* > 0.05; data not shown). Therefore, CLE or LU treatment might have a potential therapeutic effect through reduced levels of lipotoxic intermediates (LPC and CE).

Among the 63 lipid metabolites increased by the HFD, we identified 22 potential markers associated with anti-obesity effects of CLE or LU in the plasma of mice using Tukey’s multiple comparison test. In particular, the levels of 6 CE (20:2, 20:3, 20:4, 20:5, 22:4, and 22:6), 7 LPC (16:0, 18:0, 18:1, 20:2, 20:3, 20:4, and 22:6), 5 PC (34:0 (16:0/18:0), 38:4 (18:0/20:4), 38:5 (18:1/20:4), 40:6 (18:0/22:6), and 40:7 (18:1/22:6)), and 4 sphingolipid (CER 34:1 (d18:1/16:0), SM 34:1 (d18:1/16:0), SM 36:1 (d18:1/18:0), and SM 36:2 (d18:2/18:0)) were significantly lowered in the HFD group treated with CLE or LU (Supplementary Table S1). 

CER, a precursor and major molecule of sphingolipids, and SM, which may act as a pool for the generation of CER, are the most abundant sphingolipids in lipoproteins and constitute about 3% and 87% of plasma sphingolipids, respectively [55]. Similar to our study, several studies have reported that CER and SM levels were significantly increased in plasma and liver of animal models caused by a HFD or leptin deficiency [56,57,58,59]. In addition, upregulation of genes involved in sphingolipid biosynthesis has been frequently observed in obesity and dyslipidemia. The synthesis of sphingolipids is initiated by serine palmitoyl-coenzyme A (CoA) transferase (SPT) using serine and palmitoyl-CoA as substrates, and CER is generated through a two-step process by ceramide synthase (CerS) and dihydroceramide desaturase (DES1), which is then converted to SM by a transfer of the head group of PC via the action of sphingomyelin synthase (Sgms) [60]. The gene expression of the SPT isozymes, 3-ketodihydrosphingosine reductase (kdsr), CerS isozymes, and Sgms isozymes, which are involved in regulating the de novo sphingolipid biosynthetic pathway and the levels of pro-ceramide gene expression, including sptlc2, CerS6, and sgms1, were significantly increased in the HFD group compared to those in the ND group (*p* < 0.05). Similar to our results, a previous study has reported that sptlc2, which is responsible for the initial steps in sphingolipid biosynthesis, was significantly elevated in HFD-induced insulin-resistant mice relative to a low-fat diet group [61]. In addition, upregulated expression levels of ceramide synthases (CerS1, CerS2, CerS4, and CerS6) have been observed in HFD-induced insulin resistance [61,62,63].

**Figure 6:**
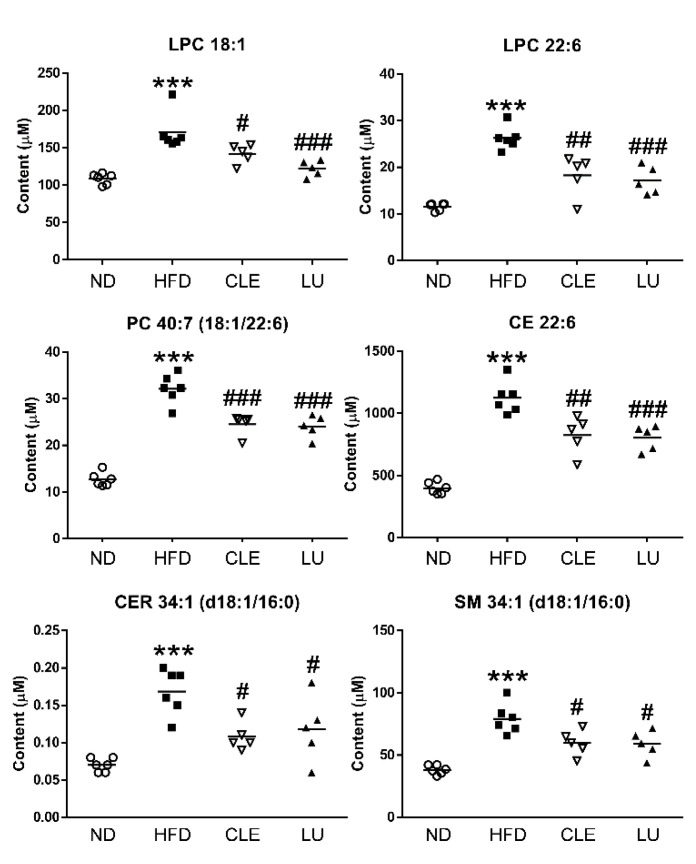
Scatter plot showing significant differences in the levels of the lipid biomarkers with the same lipid compositions among the normal diet (ND), high-fat diet (HFD), HFD plus *Chrysanthemum morifolium* Ramat leaf extract 1.5% diet (HFD + CLE), and HFD plus luteolin 0.003% diet (HFD + LU) groups. Each symbol represents an individual mouse (Circle (○): ND; Box (■): HFD; Inverted triangle (▽): CLE; Triangle (▲): LU; Diamond (◆): Quality control (QC)). *, # different letters indicate significant difference compared with the normal diet (ND) group (*) and the HFD group (#), as determined by Tukey’s multiple comparisons test. (*** *p* < 0.001, # *p* < 0.05, ## *p* < 0.01, ### *p* < 0.001). LPC: lysophosphatidylcholine; PC: phosphatidylcholine; CE: cholesteryl ester; CER: ceramide; SM: sphingomyelin.

In the present study, CLE or LU treatment markedly decreased the levels of sphingolipids (CER and SM) in plasma (Figure 5b). As a result, the hepatic mRNA expression levels of CerS4, CerS6, Sgms1, and Sgms2 were significantly reduced after CLE or LU treatment (*p* < 0.05) (Figure 5b). CER 34:1 (d18:1/16:0) synthesized by CerS6 is specifically distinguished as a key CER compared to other CERs in obesity-related diseases, which worsens the overall health status through disruption of insulin signaling, induction of apoptosis, and suppression of energy expenditure or fatty acid oxidation [63,64,65,66]. Similar to our study (Figure 6), Raichur et al. also showed that treatment with antisense oligonucleotides as a selective CerS6 inhibitor prevented the development of obesity and T2D through improved insulin sensitivity and reduced body weight gain, together with approximately a 50% reduction of CER 34:1 (d18:1/16:0) levels in both the plasma and liver [64]. In addition, sgms1 gene knockout mice showed dramatic attenuation of SM levels in plasma and liver with suppression of inflammatory cytokines [67]. Additionally, some studies reported that Sgms2 knockout could prevent the development of liver steatosis [68,69]. These results indicated that CLE or LU treatment ameliorated HFD-induced dyslipidemia in mice by lowering the lipotoxic intermediates (CER and SM) in the liver and plasma through inhibition of de novo synthesis of sphingolipids.

Taken together, our study demonstrated that treatment with CLE significantly reduced lipotoxic intermediates, such as PC, LPC, CER, SM, and CE, in the plasma of HFD-induced obesity and dyslipidemia model. Especially, six metabolites, LPC 18:1, LPC 22:6, PC 40:7, CE 22:6, CER 34:1, and SM 34:1, might be used as biomarker candidates which could explain the pharmacological effects of CLE or LU on dyslipidemic mice (Figure 6). Elevated hepatic expression levels of genes involved in the Kennedy pathway and sphingolipid metabolism related to synthesis of lipotoxic intermediates in the group of HFD-induced obese mice were dramatically recovered to the levels of the ND group after CLE or LU treatment. Consequently, the supplementation of CLE and LU was shown to ameliorate HFD-induced dyslipidemia and these lipid biomarkers can be used to better understand obesity-related metabolic disorders and to investigate their beneficial effects associated with such disorders.

## Supplementary Materials

The following are available online at https://www.mdpi.com/2072-6643/12/10/2973/s1, Figure S1: Effect of *Chrysanthemum morifolium* Ramat leaf extracts (CLE) and Luteolin (LU) supplements for 16 weeks on individual TAG species in the liver. *, # different letters indicate significant difference compared with normal diet (ND) group (*) and HFD groups (#), as determined by Tukey’s multiple comparisons test (*** *p* < 0.001, # *p* < 0.05, ## *p* < 0.01, ### *p* < 0.001). Table S1: Identified potential lipid biomarkers in plasma indicating statistically significant difference among sampling groups.

## Abbreviations

**Abbreviations**	**Definition**
BHT	butylhydroxytoluene
CE	cholesteryl ester
Cept	ethanolamine phosphotransferase
CER	ceramide
CerS	ceramide synthase
Chpt	choline phosphotransferase
CLE	*Chrysanthemum morifolium* Ramat leaf extracts
CM	*Chrysanthemum morifolium* Ramat
CVD	cardiovascular disease
DAG	diacylglycerol
DES1	dihydroceramide desaturase
HFD	high-fat diet
HPLC	high-performance liquid chromatography
IS	internal standard
kdsr	3-ketodihydrosphingosine reductase
LCAT	lecithin-cholesterol acyltransferase
LC-MS/MS	liquid chromatography coupled with tandem mass spectrometry
LDL	low-density lipoprotein
LPC	lysophosphatidylcholine
LPE	lysophosphatidylethanolamine
LU	HFD plus luteolin 0.003% diet
MTBE	methyl*-tert*-butyl ether
ND	normal diet
PC	phosphatidylcholine
PCA	principal component analysis
PE	phosphatidylethanolamine
PEMT	Phosphatidylethanolamine *N*-methyltransferase
PLA_2_	phospholipase A_2_
PLS-DA	partial least squares-discriminant analysis
PPAR	peroxisome proliferator-activated receptor
QC	quality control
Sgms	sphingomyelin synthase
SM	sphingomyelin
SPT	serine palmitoyltransferase
SRM	selected reaction monitoring
TAG	triacylglycerol
TNF	tumor necrosis factor
T2DM	type 2 diabetes mellitus
WAT	white adipose tissue
