# Supplementary figures and images for: Plasma Lipidomics Reveals Insights into Anti-Obesity Effect of Chrysanthemum morifolium Ramat Leaves and Its Constituent Luteolin in High-Fat Diet-Induced Dyslipidemic Mice
Source: Nutrients. 2020 Sep 29;12(10):2973. doi: 10.3390/nu12102973 (PMC7650530; doi:10.3390/nu12102973)

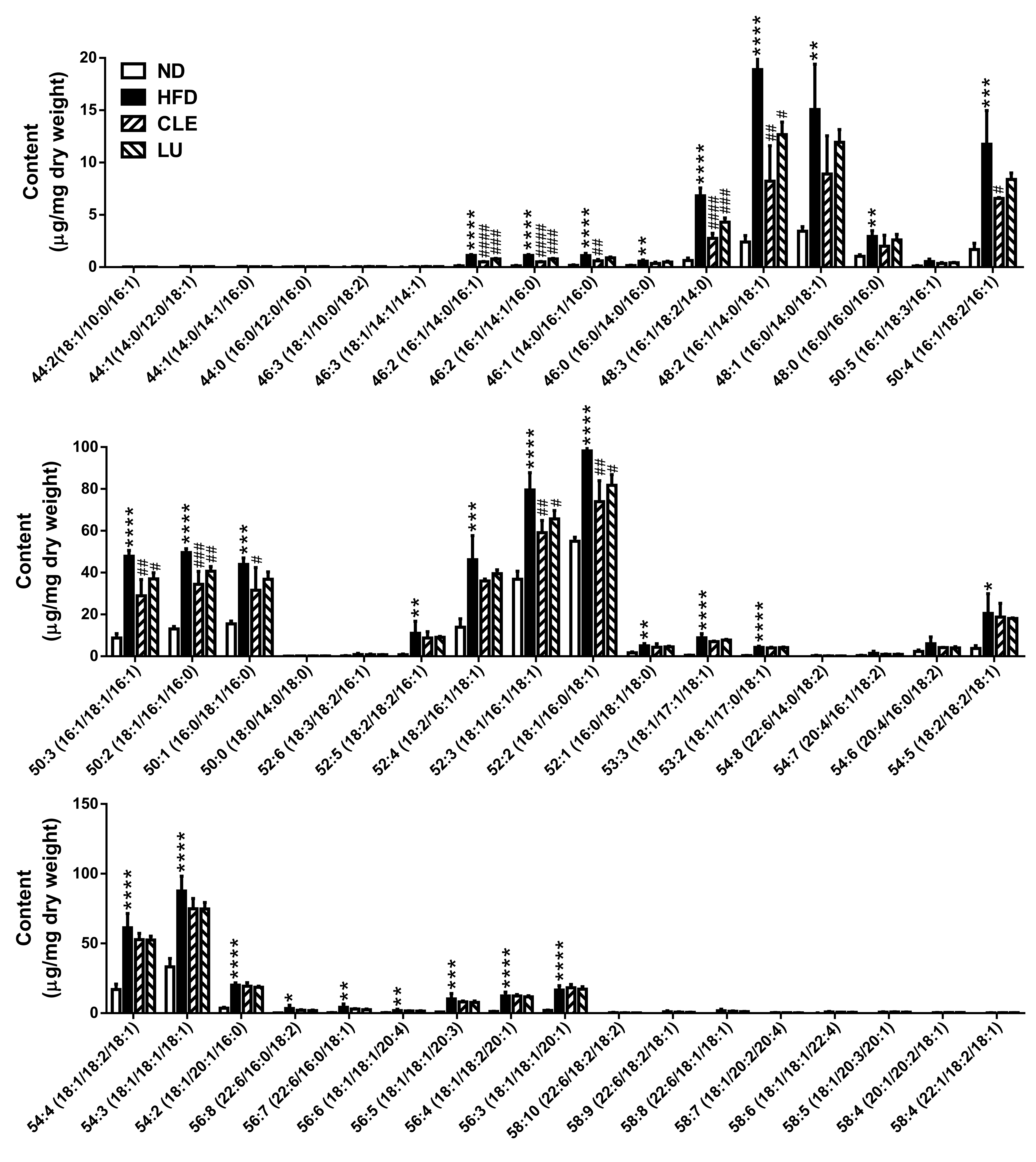

Supplement: Supplementary file 1 [file nutrients-12-02973-s001.zip › Figure S1.tif]
